# Early outgrowth cells versus endothelial colony forming cells functions in platelet aggregation

**DOI:** 10.1186/s12967-015-0723-6

**Published:** 2015-11-09

**Authors:** Lara Bou Khzam, Olivier Bouchereau, Rahma Boulahya, Ahmed Hachem, Younes Zaid, Haissam Abou-Saleh, Yahye Merhi

**Affiliations:** Laboratory of Thrombosis and Hemostasis, Montreal Heart Institute, 5000 Belanger, Montreal, QC H1T 1C8 Canada; Department of Biochemistry, Weill Cornell Medical College in Qatar, Doha, Qatar; Division of Cardiology, Sidra Medical and Research Center, Doha, Qatar; Faculty of Medicine, Université de Montréal, Montreal, QC Canada

**Keywords:** Endothelial progenitor cells, Platelets, Aggregation, Prostacyclin, Nitric oxide

## Abstract

**Background:**

Endothelial progenitor cells (EPCs) have been implicated in neoangiogenesis, endothelial repair and cell-based therapies for cardiovascular diseases. We have previously shown that the recruitment of EPCs to sites of vascular lesions is facilitated by platelets where EPCs, in turn, modulate platelet function and thrombosis. However, EPCs encompass a heterogeneous population of progenitor cells that may exert different effects on platelet function. Recent evidence suggests the existence of two EPC subtypes: early outgrowth cells (EOCs) and endothelial colony-forming cells (ECFCs). We aimed at characterizing these two EPC subtypes and at identifying their role in platelet aggregation.

**Methods:**

EOCs and ECFCs were generated from human peripheral blood mononuclear cells (PBMCs) seeded in conditioned media on fibronectin and collagen, respectively. The morphological, phenotypical and functional characteristics of EOCs and ECFCs were assessed by optical and confocal laser scanning microscopes, cell surface markers expression, and Matrigel tube formation. The impact of EOCs and ECFCs on platelet aggregation was monitored in collagen-induced optical aggregometry and compared with PBMCs and human umbilical vein endothelial cells (HUVECs). The levels of the anti-platelet agents’ nitric oxide (NO) and prostacyclin (PGI_2_) released from cultured cells as well as the expression of their respective producing enzymes NO synthases (NOS) and cyclooxygenases (COX) were also assessed.

**Results:**

We showed that EOCs display a monocytic-like phenotype whereas ECFCs have an endothelial-like phenotype. We demonstrated that both EOCs and ECFCs and their supernatants inhibited platelet aggregation; however ECFCs were more efficient than EOCs. This could be related to the release of significantly higher amounts of NO and PGI_2_ from ECFCs, in comparison to EOCs. Indeed, ECFCs, like HUVECs, constitutively express the endothelial (eNOS)—and inducible (iNOS)—NOS isoforms, and COX-1 and weakly express COX-2, whereas EOCs do not constitutively express these NO and PGI_2_ producing enzymes.

**Conclusion:**

The different morphological, phenotypic and more importantly the release of the anti-aggregating agents PGI_2_ and NO in each EPC subtype are implicated in their respective roles in platelet function and thus, may be linked to the increased efficiency of ECFCs in inhibiting platelet aggregation as compared to EOCs.

## Background

Endothelial progenitor cells (EPCs) are believed to play a significant role in vascular biology through their implication in vascular repair, cell therapy, and regenerative medicine [1–4]. Definitive proof of the existence of circulating EPCs was provided in 1997 [5], showing that human peripheral blood contains CD34^+^ and vascular endothelial growth factor receptor 2^+^ (VEGFR2^+^) progenitor cells, which can attach to fibronectin, acquire certain characteristics of mature endothelial cells (ECs) in vitro, and contribute to neoangiogenesis in vivo. Since then, numerous cells have been employed to improve cardiovascular function, including EPCs, bone marrow mononuclear cells, myocardial stem cells, mesenchymal stem cells, and embryonic stem cells [6, 7]. Among these, EPCs have emerged as important modulators in vascular biology and hemostasis. However, controversies still exist as to their therapeutic and diagnostic use [8–10]. One important aspect of EPC biology is their interaction with vascular and blood cells, which can largely influence their functional properties. More specifically, the interaction of EPCs with platelets provides the critical signal to ensure their migration and homing at sites of vascular injury and their differentiation into ECs [11–17]. Conversely, EPCs affect platelet function [18–22] and modulate endothelial repair [23–27], which add new insights to the importance of EPCs in vascular hemostasis.

In our attempt to elucidate the functional importance of EPC interactions with platelets, we have previously shown that a heterogeneous population of EPCs, derived from PBMCs after 10 days of culture, modulates the function of platelets [18], through binding to platelets via P-selectin [28]. This interaction impairs the function of platelets through an increase in cyclooxygenase-2 (COX-2) expression and prostacyclin (PGI_2_) release. Recent experimental evidence has established that cells originally defined as EPCs and used for potential therapy are not true endothelial progenitors [8, 29–31]. Indeed, recent analyses have revealed that, depending on how EPCs are isolated and cultured, the EPC population can either consist of a minimally proliferative myeloid-monocytic cell population (early EPCs, monocytic EPCs, EOCs, or cultured angiogenic cells) or consist primarily of a highly proliferative non-myeloid EC population (late EPCs, ECFCs, or late outgrowth cells) [9, 32–34]. In this regard, we have been characterizing and studying the well-defined EOC subtype and showed that they produce matrix metalloproteinase-9 and reactive oxygen species that influence their pro-angiogenic [23] and anti-platelet functions [19], respectively. On the other hand, ECFCs are considered highly proliferative cells that incorporate at sites of vascular lesions, differentiate into ECs and contribute to endothelial repair [9, 32, 33, 35]. Their anti-platelet properties could be of great importance in vascular hemostasis and repair. Accordingly, this study was designed to describe the characteristics of EOCs and ECFCs, and to compare their effect on platelet function by defining the underlying vasoactive substances implicated in this process. The major findings are that ECFCs are more efficient in inhibiting platelet aggregation than EOCs. This was related to an increase in the production of the potent anti-platelet agents PGI_2_ and NO by ECFCs, which induce significantly stronger inhibitory effects of platelet aggregation.

## Methods

### Culture and characterization of human EOCs and ECFCs

This study was carried out according to a protocol accepted by the Montreal Heart Institute ethical committee in agreement with the Declaration of Helsinki. Informed consent was obtained from healthy volunteers aged between 20 and 60 years and medication free over 10 days prior to blood sampling. Ficoll–Paque (GE Healthcare, Piscataway, NJ, USA) density gradient centrifugation was used to isolate peripheral blood mononuclear cells (PBMCs) from 100 mL of peripheral blood, as previously described [18]. To generate EOCs, 1 × 10^6^ PBMCs per cm^2^ were seeded on 6-well fibronectin-coated tissue culture plates (BD Biosciences, Mississauga, ON, Canada) in complete endothelial growth media EGM-2 (Lonza Inc., Burlington, ON, Canada) at 37 °C in an atmosphere of 5 % CO_2_. Following 3 days of culture, the medium was changed to remove non-adherent cells. Additional culturing of adherent cells up to 7 days was allowed to obtain EOCs [23, 36]. ECFCs were generated from PBMCs seeded at a density of 5 × 10^6^ mononuclear cells per cm^2^ in 6-well collagen-coated tissue culture plates (BD Biosciences). Culturing of ECFCs was performed in EGM-2 supplemented with 10 % FBS. The medium was changed daily for 7 days and every other day thereafter until the appearance of endothelial cell colonies (2–4 weeks), which were cultured until the formation of a confluent monolayer of highly proliferative EPCs [36–38]. Cells were used between passages three and six. Characterization of EOCs and ECFCs was compared to mature human umbilical vein endothelial cells (HUVECs) (Lonza Inc.) cultured in complete EGM2 with 10 % FBS on 0.2 % gelatin-coated flasks.

### Confocal microscopy

EOCs and ECFCs were incubated with DiI-labeled acetylated low density lipoprotein (DiI-Ac-LDL) (Invitrogen, Carlsbad, CA, USA) for 2 h followed by 4 % paraformaldehyde fixation and subsequent 1-hour incubation with FITC-labeled Ulex-lectin (Sigma-Aldrich, St-Louis, MO, USA). Nuclear staining was done with TO-PRO-3 (Invitrogen). Cells were visualized under an LSM 510 confocal microscope (Zeiss, Oberkochen, Germany) at 63× objective magnification [18].

### Matrigel tube formation assay

EPC’s potential to form tube-like structures was compared to that of HUVECs on Matrigel-coated 24-well plates [39]. Growth factor reduced Matrigel (BD Biosciences) (200 μL) was incubated for 30 min at 37 °C in a 5 % CO_2_ atmosphere to allow polymerization and formation of a gel-like surface. Cells were harvested with trypsin–EDTA 0.05 % (Gibco, Burlington, ON, Canada), washed and resuspended in complete EGM-2 at 200 × 10^3^ per 300 μL per well. Following 24-h incubation at 37 °C in a 5 % CO_2_ atmosphere, cells were viewed under an inverted microscope and images were taken at 10× objective magnification.

### Cell surface marker expression

Phenotypic characterization of EOCs and ECFCs was performed by flow cytometry and compared to PBMCs and HUVECs [18]. Cells were harvested by washing cells with phenol red-free and serum-free basal RPMI 1640 medium followed by trypsinization with 0.05 % trypsin–EDTA (Gibco) for 15 min at 37 °C [38]. Cells were subsequently blocked with normal mouse serum for 15 min, washed and incubated for 30 min with PE-labeled fluorescent human monoclonal antibodies against the monocytic marker CD14 (R&D Systems, Minneapolis, MN, USA), the leukocytic marker CD45 (AbD Serotec, Oxford, UK), the progenitor marker CD34 (BD Biosciences), the angiogenic marker VEGFR2 (R&D Systems), and the endothelial markers CD31 (BD Biosciences) and CD144 (BD Biosciences). Cells were then fixed with paraformaldehyde for 30 min and analyzed on an Altra flow cytometer (Beckman Coulter, Mississauga, ON, Canada). Cells were gated by their characteristic forward and side scatter properties.

### Platelet aggregation

Platelets were isolated from 100 mL peripheral blood of healthy volunteers, as previously described [18, 19]. Washed platelets resuspended in HBSS–Hank’s buffer were then adjusted to 250 × 10^6^/mL. Platelet aggregation was performed in a four-channel optical aggregometer (Chronolog Corp., Havertown, PA, USA) under shear (1000 rpm) at 37 °C. A volume of 400 μL of the washed platelet preparation was incubated with 100 μL of PBMC, EOC, and ECFC cells or their supernatants at 37 °C for 5 min prior to the assay. Platelet aggregation was then induced with 1 μg/mL collagen (Chronolog Corp.), and traces were recorded until stabilization of platelet aggregation was reached. Controls were performed by incubating platelets with complete EGM-2 culture medium.

### Western blot

EOCs and ECFCs were harvested using trypsin–EDTA 0.05 % (Gibco), centrifuged at 500×*g* for 10 min then resuspended in PBS 1× and sonicated. Protein content was assessed by the Bradford assay method, mixed with the appropriated volume of 4× Laemmli loading buffer and heated for 5 min at 95 °C. Protein lysates (40 μg) were resolved by SDS-PAGE and transferred onto nitrocellulose membranes (Bio-Rad, Hercules, CA, USA). Membranes were blocked with 5 % non-fat milk in TBS-Tween-20 for 1 h. Membranes were then incubated overnight with primary antibodies (1:1000) against eNOS and iNOS (Cell Signaling, Beverly, MA, USA), and against COX-1 and COX-2 (Santa Cruz Biotechnology, Santa Cruz, CA, USA). Following washing steps, membranes were labeled with horseradish peroxidase-conjugated secondary antibody for 1 h, washed and bound peroxidase activity was detected by enhanced chemiluminescence (Perkin Elmer Life Sciences, Waltham, MA, USA).

### PGI_2_ release assay

A commercial radioimmunoassay (RIA) PGI_2_ kit was used to assess prostacyclin release (Assay Designs, San Diego, CA, USA) according to manufacturer instructions. Levels of 6-keto-PGF_1∝_, the stable metabolite of PGI_2_, were assessed in EOC and ECFC culture supernatants and compared to PBMC and HUVEC supernatants.

### NO release assay

A commercial fluorometric NO assay kit was used to assess nitric oxide release (Cayman Chemicals, Ann Arbor, MI, USA) according to manufacturer instructions. Levels of total nitrate and nitrite (NO_2_^−^ and NO_3_^−^) were assessed in EOC and ECFC supernatants and net nitrate (NO_2_^−^) concentrations were calculated and compared to PBMC and HUVEC supernatants.

### Statistical analysis

Results are presented as mean ± SEM of at least three independent experiments. Statistical comparisons were done using either paired student’s *t* test or a one-way ANOVA followed by a Dunnett’s-*t*-test for comparison against a single group. Data with *p* < 0.05 were considered statistically significant.

## Results

### EOCs and ECFCs are morphologically and phenotypically different

**Fig. 1:**
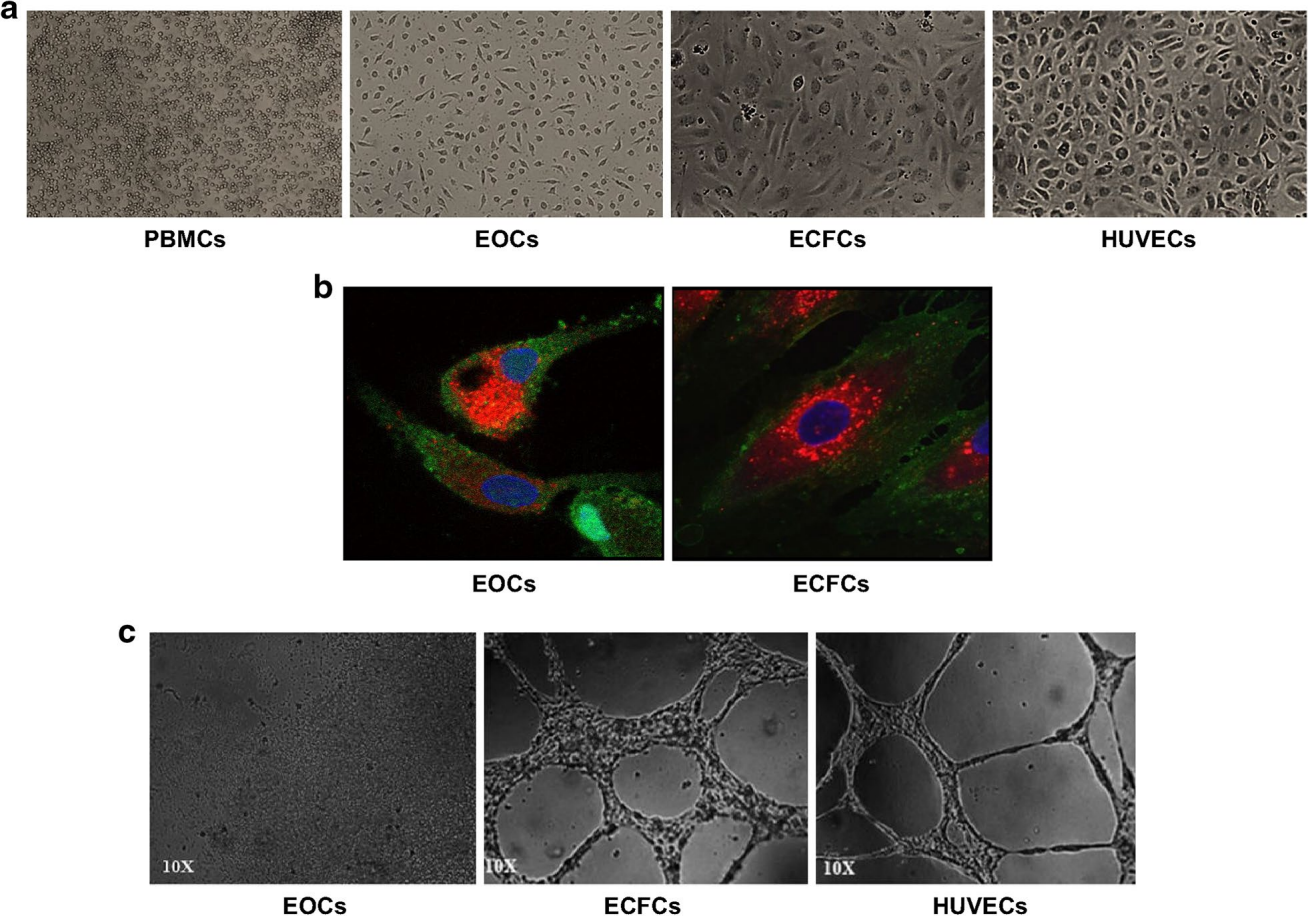
Characterization of PBMC-derived EOCs and ECFCs. **a** Representative optical microscopy images of EOCs and ECFCs taken at 10× magnification using an inverted light microscope. EOCs display a heterogeneous population of round and elongated cells on fibronectin following 7 days of culture. ECFCs show a homogeneous population forming a cobblestone-like monolayer on collagen following 21 days of culture. **b** Representative confocal microscopy images showing EOCs and ECFCs triple staining for DiI-Ac-LDL uptake (*red*), Ulex-lectin binding (*green*) and TO-PRO-3 nuclear staining (*blue*) taken at 63× magnification. **c** Representative optical microscopy images showing the tube-like structure formation potential of EOCs and ECFCs compared to HUVECs on a Matrigel surface taken at 10× magnification using an inverted microscope

Different culture techniques were required to generate each EPC subtype. EOCs were generated from PBMCs cultured for 7 days on fibronectin-coated plates whereas ECFCs were obtained following longer culture periods (2–4 weeks) on collagen-coated plates. The morphology observed for each cell subtype differs greatly. EOCs form a heterogeneous population of round and elongated cells whereas ECFCs form a cobblestone-like monolayer of homogenous appearance (Fig. 1a). EOCs display an immature endothelial cell character since they still resemble the PBMC population from which they derive following 7 days of culture. On the other hand, ECFCs seem to acquire an endothelial-like phenotype resembling HUVECs. Furthermore, EOCs are short-lived cells which do not survive past 7 days whereas ECFCs are highly proliferative cells which can be passaged and kept in culture for months (data not shown). Both EOCs and ECFCs bind Ulex-lectin and internalize DiI-Ac-LDL, both endothelial cell characteristics (Fig. 1b).

### EOCs and ECFC tube formation potential

We show that EOCs and ECFCs are also functionally different. EOCs do not form tube-like structures, whereas ECFCs, similarly to HUVECs, form well-structured tubes on Matrigel (Fig. 1c).

### EOCs and ECFC cell surface marker expression

**Fig. 2:**
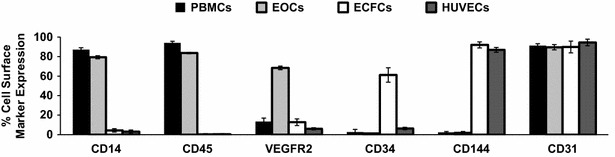
Expression of cell surface markers by EOCs and ECFCs. Flow cytometric analysis of cell surface marker expression profile of EOCs (*gray*) and ECFCs (*white*) compared to PBMCs (*black*) and HUVECs (*dark gray*) using mouse anti-human PE-conjugated monoclonal antibodies against CD14, CD45, VEGFR2, CD34, CD144 and CD31. *Histogram* represents the mean data ± SEM of percent cell surface marker expression (*n* ≥ 3)

EOCs continue to express the leukocytic and monocytic cell surface markers CD45 and CD14, respectively, after 7 days of culture, whereas ECFCs lose these markers throughout their differentiation process. Moreover, EOCs strongly express the angiogenic marker VEGFR2. Unlike EOCs, ECFCs express the progenitor cell marker CD34 and the endothelial cell marker CD144. All cell populations showed uniform expression of the platelet-endothelial cell adhesion molecule CD31 (Fig. 2).

### EOCs and ECFCs inhibit platelet aggregation

**Fig. 3:**
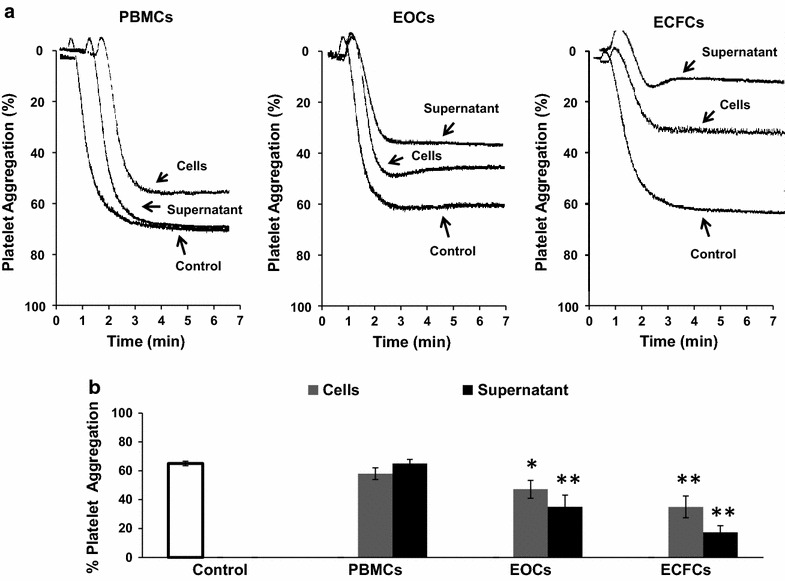
Effect of PBMCs, EOCs and ECFCs on platelet aggregation. **a** Representative traces of platelet aggregation. Platelets were incubated with complete EGM-2 medium (control) or in the presence of 4 × 10^6^/mL PBMC, EOC and ECFC cells or their respective supernatants for 5 min at 37 °C prior to aggregation initiation with 1 μg/mL of collagen. **b** *Histogram* represents the mean data of percent platelet aggregation ± SEM (*n* ≥ 3, ** *p* < 0.01 vs control)

In order to better understand the influence of EPCs on platelets, we sought to investigate the effect of each EPC subtype on platelet aggregation. We show that both EOCs and ECFCs and their respective supernatants inhibit platelet aggregation, in comparison to PBMCs (Fig. 3). However, we noticed an increased efficiency of the supernatants in inhibiting platelet aggregation in comparison to their respective cell samples for both EOCs and ECFCs. We suggest that this may be due to the release of vasoactive agents which play an important role in platelet aggregation. Furthermore, we demonstrated that ECFCs are more efficient at inhibiting platelet aggregation than EOCs.

### ECFCs release more NO and PGI_2_ than EOCs

**Fig. 4:**
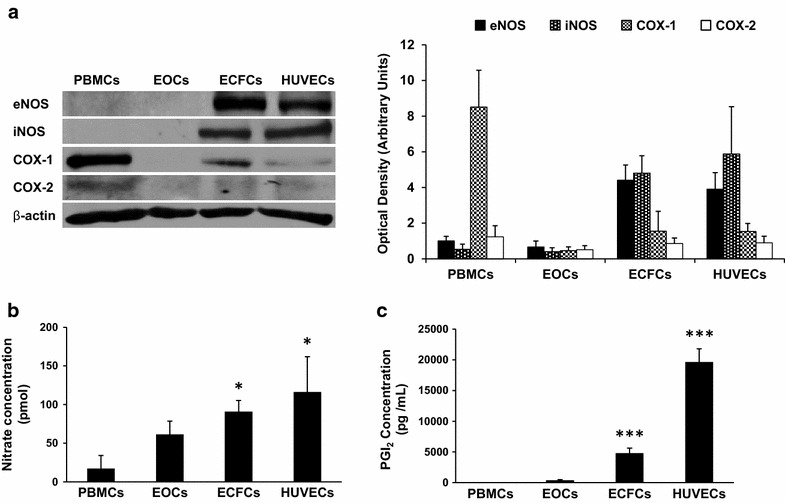
Expression of NOS and COX and release of NO and PGI_2_ by EOCs and ECFCs. **a** *Left*: NOS and COX protein expression profile in PBMCs, EOCs, ECFCs and HUVECs. Representative blots of cell lysates analyzed for eNOS, iNOS, COX-1 and COX-2 by SDS-PAGE. *Right*: *Histogram* represents the mean of data ± SEM expressed as arbitrary units of optical density of blots on the left (*n* ≥ 3); (**b**, **c**) supernatants from 4 × 10^6^/mL PBMCs, EOCs, ECFCs and HUVECs were assessed for PGI_2_ release by radioimmunoassay of 6-keto-PGF_1α_ concentration (pg/mL) (**b**) and for NO release by nitrate/nitrite fluorometric assay for nitrate concentration (pmol) (**c**). Histograms represent the mean concentration ± SEM of nitrate and PGI_2_ (*n* ≥ 3, *p < 0.05 vs PBMCs, ***p < 0.001 vs PBMCs)

To further understand the increased efficiency of ECFCs compared to EOCs in inhibiting platelet aggregation, we compared the NOS and COX expression profiles in EOCs, ECFCs, PBMCs, and HUVECs. We found that ECFCs, similarly to HUVECs, constitutively express eNOS, iNOS, and COX-1, but to a lesser extent COX-2. In contrast, EOCs do not constitutively express these NO and PGI_2_ producing enzymes (Fig. 4a). Using radioimmunoassay and fluorometric techniques, we assessed PGI_2_ and NO release, respectively. We found that EOCs release minimal amounts of NO (60 pmol) and PGI_2_ (300 pg/mL) whereas ECFCs, similarly to HUVECs, release significantly higher levels of both NO (90 pmol) and PGI_2_ (5000 pg/mL) (Fig. 4b,c). The higher expression of NOS and COX enzymes in ECFCs account for the significantly higher levels of NO and PGI_2_ released by these cells in comparison to EOCs.

## Discussion

EPCs have gained much attention in the past decade for their therapeutic role; however, no clear consensus has been achieved to date as to the origin and specific function of these cells. Indeed, the term EPCs represents a complex assortment of progenitor cells that play different role on neoangiogenesis and vascular repair. The characterization and critical re-evaluation of EPC phenotypes are therefore essential for their efficiency as therapeutic and diagnostic tools (for review Fadini et al. [9]). Different conditions such as source, media, incubation times and culture surfaces have yielded different subtypes of EPCs. However, recent evidence suggests the existence of two subtypes of EPCs according to their time-dependent appearance. Based on new findings and on standardized isolation and differentiation protocols [30, 34, 36–38], we have first successfully differentiated two subtypes of EPCs from PBMCs: EOCs and ECFCs, each distinct in their morphology, phenotype, and function. As it has previously been argued, we have found that EOCs and ECFCs differ in morphology and phenotype since EOCs possess monocytic-like characteristics, whereas ECFCs are rather endothelial-like cells. In addition, EOCs neither proliferate nor form tube-like structures, whereas ECFCs are highly proliferative and form structured and defined tubes on Matrigel. These data confirm previous studies showing that EOCs lack the ability to incorporate into the neovasculature, but rather influence neovascularization through the release of paracrine factors, whereas ECFCs can be incorporated in new vessel formation [36, 40]. However, the two EPC subtypes seem to act synergistically in vascular repair [40–42]. Therefore, we aimed at studying the role of EOCs and ECFCs in platelet function as the former cannot form new vessels but may considerably influence platelets by releasing a multitude of vasoactive substances, whereas the latter may indirectly influence platelet function while incorporating at sites of vascular lesions.

EPCs and platelet interactions occur at sites of vascular lesions in order to promote vascular repair. Being the first cells to be recruited at sites of vascular damage, platelets become activated to release a multitude of paracrine factors (SDF-1) and to express cell surface adhesion molecules (P-selectin), which allow for the recruitment and differentiation of EPCs [14, 15, 43]. In turn, EPCs also modulate the function of platelets by releasing paracrine factors [18, 19], such as PGI_2_ and NO, which are the two main endothelium-derived vasodilation substances that display potent anti-platelet properties [44]. In the present study, we showed that both EOCs and ECFCs inhibit platelet aggregation. However, ECFCs are significantly more efficient than EOCs at inhibiting this process. Interestingly, the supernatants from both EOCs and ECFCs inhibit platelet aggregation more efficiently than their respective cells. Next, we compared the levels of PGI_2_ and NO released by EOCs and ECFCs, as these two substances are the major endothelium-derived vasodilation agents that play protective and antithrombotic roles in platelet reactivity. We showed that EOCs release smaller quantities of both PGI_2_ and NO in comparison to ECFCs. This could be related to the presence of a higher quantity of NO and PGI_2_ in the supernatants, which accounts for their increased efficiency in inhibiting platelet function as compared to the cells. In this connection, COX-1 and COX-2 are the enzymes involved in the production of prostaglandins, thromboxane and PGI_2_. Nitric oxide synthases (eNOS, iNOS and nNOS) are the enzymes involved in NO production, among which eNOS and iNOS are mainly responsible for NO production by the endothelium. Different levels of expression and regulation of the COX and NOS enzymes determine the levels of PGI_2_ and NO released by different vascular cell types. Interestingly, we found that ECFCs, similarly to HUVECs, constitutively express eNOS, iNOS and COX-1, but to a lesser extent COX-2. Surprisingly, EOCs do not constitutively express these NO and PGI_2_ producing enzymes. This could be related in part to a regulatory mechanism that occurs at this early stage of the differentiation process, where EOCs, like other NO and PGI_2_ producing cells, need to be triggered to upregulate COX and NOS enzymes. However, further investigation is needed to clarify this issue. In the other hand, the pattern expression of COX and NOS in our ECFC population was close to those found in EPCs generated in our previous study [18]. This could explain why ECFCs release considerably higher amounts of the anti-aggregating agents PGI_2_ and NO than EOCs, thereby accounting for their enhanced capacity in inhibiting platelet aggregation. However, the mechanism by which EOCs and their supernatants inhibit platelet aggregation remains to be determined. In addition, further research is required to understand the intracellular mechanisms underlying the differential influence of EOCs and ECFCs on platelet function.

## Conclusions

Endothelial progenitor cells are regarded as promising therapeutic tools for vascular repair, tissue engineering, and regenerative medicine. However, the clinical usefulness of these cells is hindered by the existence of diverse subtypes of EPCs which exert diverse therapeutic potential. EPCs generated from the culture of PBMCs have been identified as EOCs and ECFCs depending on the culture conditions. In this study, we determined the characteristics of EOCs and ECFCs, and we defined their impact on platelet aggregation, the essential function in both vascular injury and vascular repair. We found that EOCs and ECFCs display distinct morphological, phenotypical, and functional characteristics. In addition, we demonstrated that both EOCs and ECFCs inhibit platelet aggregation, however; the inhibitory effect of ECFCs was more pronounced than EOCs. These inhibitory effects seem to be mediated by the secretion of the anti-aggregating agents PGI_2_ and NO by EOCs and, to a greater extent by ECFCs, thus accounting for their enhanced capacity in inhibiting platelet aggregation. These findings enforce our current understandings of different subsets of EPCs and provide novel insights into the differential role played by EOCs and ECFCs in modulating platelet function during vascular repair. This knowledge would benefit the management of atherothrombosis during acute coronary syndromes and following percutaneous coronary interventions. Ultimately, this may lead to the development of novel strategies for EPC-derived antithrombotic therapies and vascular regeneration in patients with cardiovascular disease.

## Abbreviations

EPCs: endothelial progenitor cells
ECs: endothelial cells
EOCs: early outgrowth cells
ECECs: endothelial colony forming cells
PBMCs: peripheral blood mononuclear cells
HUVECs: human umbilical vein endothelial cells
VEGFR2: vascular endothelial growth factor receptor 2
PGI_2_: prostacyclin
NO: nitric oxide
COX: cyclooxygenase
eNOS: endothelial nitric oxide synthase
iNOS: inducible nitric oxide synthase
